# Assessing the impact of a community-based psychodrama intervention on mental health promotion of adolescents and young adults in Mozambique: A mixed-methods study

**DOI:** 10.7189/jogh.14.04182

**Published:** 2024-07-26

**Authors:** Roberto Benoni, Chiara Malesani, Anna Sartorello, Hamilton Cardoso, Izilda Chaguruca, Chivite Alamo, Giovanni Putoto, Giulia Brigadoi, Annachiara Zin, Daniele Donà, Carlo Giaquinto, Michela Gatta

**Affiliations:** 1 Division of Pediatric Infectious Diseases, Department of Women’s and Children’s Health, University of Padua, Padua, Italy; 2 Doctors with Africa CUAMM, Beira, Mozambique; 3 Section of Hygiene, Department of Diagnostics and Public Health, University of Verona, Verona, Italy; 4 Child and Adolescent Neuropsychiatry Unit, Department of Women's and Children's Health, Padua University Hospital, Padua, Italy; 5 Section of Operational Research, Doctors with Africa CUAMM, Padova, Italy

## Abstract

**Background:**

While mental health is an emerging issue in low-income countries, its promotion remains poor, with little context-oriented evidence available. Here we aimed to assess the impact and acceptability/feasibility of a community-based psychodrama intervention involving both adolescents and young adults (AYA) living with HIV (AYALHIV) and without HIV (AYAHIV−).

**Methods:**

We used a mixed-methods approach, where the quantitative part was based on pre/post questionnaires and the qualitative one on content analysis of semi-structured interviews. Trained community health care workers delivered psychodrama sessions involving AYAs aged 15–24 years once a week between July and August 2023 in Beira, Mozambique. The baseline assessment involved a sociodemographic and three mental health questionnaires: the Mental Health Knowledge Schedule (MAKS), Community Attitudes toward People with Mental Illness (CAMI27), and the Toronto Alexithymia Scale (TAS). We repeated all questionnaires after the intervention and conducted semi-structured interviews.

**Results:**

We enrolled 354 AYAs (50.8% female, 45.5% AYALHIV) at baseline; 315 (89.0%) attended the sessions, with a mean participation rate of 94.4%. Overall, 281 (89.2%) completed the post-intervention assessment. MAKS score improved from 44.5 (95% confidence interval (CI) = 44.0–45.0) to 47.1 (95% CI = 46.4–47.7, *P* < 0.001). Total CAMI27 score showed no significant variation (*P* = 0.855). Total TAS score decreased from 57.3 (95% CI = 56.1–58.5) to 54.3 (95% CI = 53.0–55.6, *P* < 0.001). We found no statistically significant interactions between time and sex, age, or HIV serostatus in all questionnaires. For the qualitative part of the study, we analysed 37 semi-structured interviews (43.2% with females, 40.5% with AYALHIV). We identified four areas of intervention impact: peer-to-peer support (30.3%), social empowerment (24.7%), resilience (23.0%), and emotional skills (21.9%). Regarding acceptability/feasibility, perceived scalability (37.2%) and affective attitude (34.5%) were the sub-areas most frequently retrieved in the SSIs.

**Conclusions:**

The community-based psychodrama intervention proved to be an effective tool in AYAs' mental health promotion, increasing knowledge and improving emotional awareness through group experience and interpersonal learning. The intervention also showed good acceptability and feasibility in the context of our study.

Like most sub-Saharan African (SSA) countries, the population of Mozambique mainly comprises adolescents and young adults (AYA), with 22.4% being aged between 15 and 24 years [1]. This period of life, characterised by psychophysical and social changes and challenges, brings a high risk for the onset of mental health illness; for example, up to half of all mental health disorders start before the age of 14, while suicide is the third leading cause of death in adolescents aged 15–19-year-old [2,3].

Mozambique is particularly affected by mental health problems. It has an estimated suicide rate of 13.7 per 100 000 population, more than double the average for the African region (6.0 per 100 000 population), and is the sixth country with the highest suicide rate in the African region [4]. Data from 2018 on mental health diseases in Mozambique show a prevalence of 54% of AYAs being positive on at least one screening tool for anxiety, depression, PTSD, or drug-alcohol abuse [5]. Notably, Mozambique also has the eighth highest HIV prevalence globally [5]. HIV has been recognised as a major risk factor for the development of mental health problems, with the prevalence of positive screening for mental health disorders increasing to 73% in the group living with HIV [5].

While it is well recognised that adolescence is a period of vulnerability, the World Health Organization (WHO) points out that this age also offers great potential for mental health promotion and prevention [6,7]. Such activities could enable AYAs to realise their potential and participate meaningfully in their communities [6]. Among the main targets of the WHO Guidelines on mental health promotive and preventive interventions for adolescents are psychosocial interventions aimed at helping AYAs develop skills that may positively influence their behaviour, thoughts, feelings, and social interactions [7]. Fostering socioemotional competencies and improving mental health knowledge has been shown to be one of the most effective approaches to reducing stigma around mental health problems and promoting help-seeking behaviours, making it a key strategy for preventing mental disorders, risky behaviour, and suicide [8-10].

Community-based interventions in low- and middle-income countries (LMICs) have proved to be effective in improving mental health and reducing violence and substance use in AYAs [11]. Among the interventions implemented to promote psychological well-being of individuals and communities, psychodrama has widely been used in diverse settings and for various issues, including stress related to HIV-positive diagnosis [12]. Created by Jacob Levy Moreno in 1921, psychodrama is a group format therapeutic model with deep roots in theatre, psychology, and sociology [13]. It offers participants the opportunity to explore feelings, thoughts, and behaviours through actions rather than words alone [14]. Psychodrama aligns with positive psychology by focussing on promoting people's capacities to actively participate in healing themselves and thriving, both as individuals and as a society [15]. Although psychodrama has proven to be an effective tool in both therapeutic and educational settings, few studies evaluated the effectiveness of related interventions among AYAs in the context of resource-limited countries [15]. Thus, we aimed to assess the impact of a community-based psychodrama intervention on AYAs’ knowledge and attitudes towards mental health, and their emotional consciousness and skills, as well as to consider possible differences in this impact based on the HIV serostatus. Our secondary objectives were to evaluate the acceptability and feasibility of this intervention and to describe the baseline level of mental health knowledge and attitudes of AYAs living with (AYALHIV) and without HIV (AYAHIV−). We hypothesised that a community psychodrama intervention could be associated with increased knowledge about mental health and improved social-emotional skills of AYAs being equally effective in AYALHIV and AYAHIV− working in mixed groups.

## METHODS

### Ethical approval

We performed this study per the 1964 Declaration of Helsinki, and we received approval from the Comité Interinstitucional de Bioética para Saúde (CIBS) – Sofala on the 21 July 2023 (protocol number 007/CIBS/2023).

### Study design, setting, and population

This was a mixed-methods study, where the quantitative analysis was based on data from pre-post questionnaires, and the qualitative one on semi-structured interviews. We carried out this study in the Beira, province of Sofala, Mozambique, between July and September 2023.

We recruited AYAs at the community level in four neighbourhoods, and AYALHIV specifically from the adolescents' and youths' friendly service (*serviço amigo dos adolescentes e jovens* (*SAAJ*)) of the four health centres within these neighbourhoods. These services provide education, prevention, primary care support, and HIV treatment for target population aged between 10 and 24 years.

We considered all persons aged between 15 and 24 years living in the four neighbourhoods or accessing the *SAAJ* during the study period as eligible for inclusion. For AYALHIV, we excluded those who had been diagnosed less than three months before the study period. We otherwise excluded AYAs attending less than five (65%) sessions.

### Participant and public involvement

We involved local health authorities and key stakeholders at the community level in setting priorities for the scope of action and planning the intervention. We otherwise sought the participants’ personal feedback on the intervention in the qualitative part of our study.

### Psychodrama intervention

This intervention encompassed three phases. First, we provided training for eight community health workers (CHW) from a local cooperative. It started with a five-day intensive course; two days were dedicated to theory, to give the participants basic knowledge on mental health, while three days were focussed on experience-oriented training to let them experience a psychodrama session and acquiring the most important psychodrama-related principles and techniques.

Second, we created AYA groups. Here, the CHWs were divided into four pairs, each active in a different neighbourhood. They recruited AYAs for the baseline assessment and enrolled them in a list for subsequent group formation. After the baseline assessment, the CHWs called up the AYAs and formed groups with those available, resulting in 30 groups each ranging from 8 to 13 AYAs.

**Figure 1:**
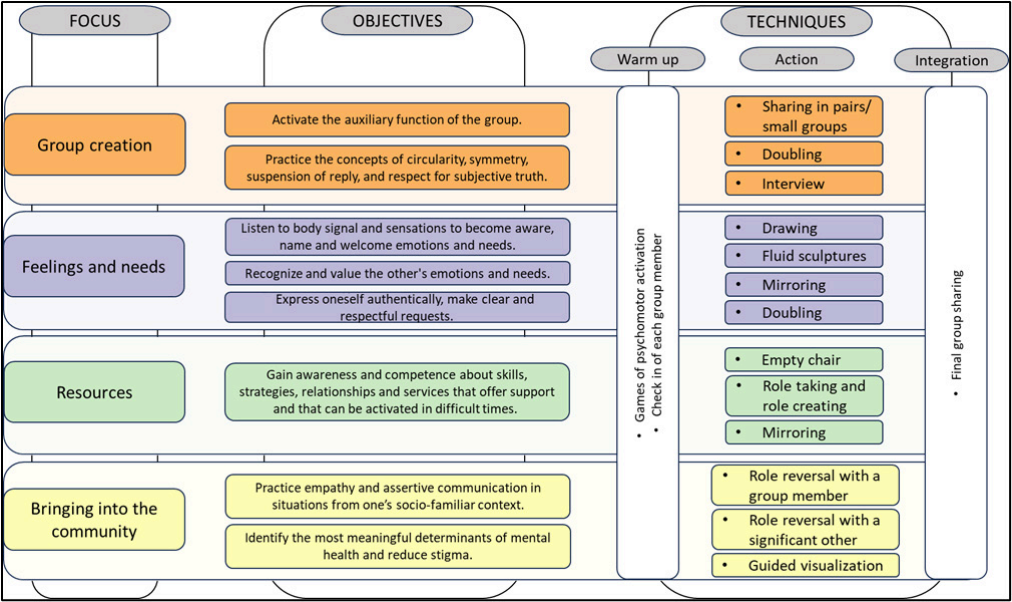
Framework of the psychodrama intervention implemented in the study.

Third, we implemented the psychodrama sessions themselves and provided ongoing training for the CHWs. For eight consecutive weeks, the CHWs took part in a weekly training, actively exploring the specific themes and techniques of each session they were going to have with the AYAs during the week. Eight weekly psychodrama sessions were conducted for each group in total. Psychodrama techniques were applied across all sessions to promote emotional self-awareness, empathy, and conflicts mediation (**Figure 1**).

### Sample size

For the pre-post questionnaire, we calculated that we needed a minimum sample of 315 with the following formula: (Z*_α_*_/2_ + Zβ)^2^ /d^2^, with α and β set at 0.05 and 0.2, respectively, and the effect size (d) set at 0.17, expressing a small effect size, and considering a lost to follow-up rate of 15% [16].

There is no consensus in literature on sample size estimation for qualitative analyses, although some authors suggest that 6 interviews would correspond to an 80% saturation of topics or 12 to a higher saturation rate [17]. To better represent our sample, we collected two interviews from each psychodrama group, resulting in a total of 60 interviews. We selected the two participants in each group randomly, drawing a number from a container corresponding to each participant (simple random sampling) from those present in the last session.

### Questionnaires

We used two questionnaires to assess knowledge and attitude towards mental health, respectively. The Mental Health Knowledge Schedule (MAKS) has 12 items constructed on a 5-point Likert scale, with a maximum total score of 60 points. A score above 35 gives a good level of mental health knowledge [18]. In turn, the 27-item’s version of the Community Attitudes toward People with Mental Illness (CAMI27) has a two factors structure: ‘prejudices and exclusion’ and ‘tolerance and support in the community.’ It has a 5-point Likert scale response scheme with a maximum total score of 135 points. Both MAKS and CAMI27 have been previously validated in Portuguese and in LMIC settings [19–21].

We used the Toronto Alexithymia Scale (TAS) to assess the impact of the psychodrama intervention on participants' perceptions about expressing and describing their emotions. It includes 20 items with a 5-point Likert scale divided into three factors: ‘identifying feeling,’ ‘describing feelings,’ and ‘externally-oriented thinking.’ The total score ranges from 20 to 100 points, with a higher score suggesting a higher level of alexithymia (cut-off: 20–51 for non-alexithymia, 52–60 for possible alexithymia, 61–100 for alexithymia) [22]. A Portuguese validated version of the TAS questionnaire is available [23].

### Data collection

At baseline, we asked all participants to complete an informed consent form; we obtained consent from parents or legal guardians if they were underaged. We administered a sociodemographic questionnaire (**Online Supplementary Document**) along with the MAKS, CAMI27, and TAS. Two CHWs and a child and adolescent neuropsychiatry resident supervised the baseline assessment to ensure that participants understood the questions and to address any problems. To ensure privacy and to match the pre- and post-tests, we anonymised the participants’ data through a numeric alpha code.

We then repeated the MAKS, CAMI27, and TAS questionnaires after the psychodrama intervention. On the same day that the post-test was applied, a single CHW who had received training in the interview methodology carried out on the semi-structured interview in the local language (Portuguese) using a question guide (**Online Supplementary Document**). The interviews were recorded and transcribed, with the transcripts analysed in Portuguese and only select quotes translated into English for presentation here in the manuscript.

### Content analysis

We performed an inductive content analysis to identify themes in the interview and codify them. First, we obtained a full overview of the transcripts, reading it repeatedly, after which we highlighted the parts of the text that may capture a theme of interest. We then reread the transcripts and labelled the themes, proceeding to group the labels that referred to similar concepts into categories. We then repeatedly continued this process of reading the transcripts and labelling/grouping the themes, until all highlighted themes were grouped into categories. Lastly, we organised the retrieved categories into a hierarchical structure (identifying areas and eventual sub-areas) based on the relationship between them, only to finally develop definitions and codes for each area and subarea.

Two researchers (CM and AS) applied these steps to six semi-structured interviews to identify possible themes and code them into areas and sub-areas. They discussed all potential classifications with a third researcher (RB) to obtain a consensus. Then, we created a classification index code which we tested on four more interview transcripts (**Online Supplementary Document**). Using this classification, two researchers (CM and AS) independently assessed and coded the remaining transcripts, with codes and excerpts presented below in this manuscript. We discussed discrepancies in the coding process and resolved them by involving a third rater (RB).

### Statistical analysis

We first conducted a descriptive analysis, where we described categorical variables through frequencies and percentages and continuous variables through means and standard deviations (SDs) or medians and interquartile ranges (IQRs). We assessed for differences in the participants’ sociodemographic characteristics through the χ^2^ test, Fisher exact test, and Wilcoxon-signed-rank test. We used the Shapiro-Wilk test to test the normality of the distribution of the included variables.

We measured the participants’ socioeconomic status (SES) by considering household factors based on the available literature [24,25]. Afterwards, we built an SES index using principal component analyses of the abovementioned variables, categorised into wealth tertile (low, middle, high) [26].

We explored the association between baseline score of the MAKS, CAMI27, and TAS and sociodemographic characteristics using the analysis of covariance (ANCOVA) test. To assess the change in the total score of the three questionnaires, we used the repeated-measures analysis of variance (RMANOVA) test, imputing one within-subject factor (time pre/post) and three between-subject factors (sex, age, and HIV serostatus). We otherwise used multivariate analysis of variance (MANOVA) with repeated measure to assess differences in the scores of the two and three factors of the CAMI27 and TAS questionnaires, respectively.

To identify the issues the participants’ discussed more frequently, we performed a frequency count of all areas and sub-areas retrieved in interviews. We calculated Cohen’s kappa values to assess the agreement between the two raters on the four SSIs used to test the classification index code, where agreement was considered satisfactory if the values ranged between 0.61 and 1.00 (from moderate to almost perfect level of agreement) [27]. We used the χ^2^ test to assess differences in the frequency of the different areas according to sex, age, and HIV serostatus.

A *P*-value <0.05 denoted statistical significance. We performed all analyses in R, version 4.1.1 (R Core Team, Vienna, Austria).

## RESULTS

### Baseline assessment

Overall, 354 AYAs attended the baseline assessment; 188 (53.1%) were adolescent (aged between 15 and 19 years) and 180 (50.8%) were females. There were 161 AYALHIV (45.5%), with no differences based on sex (*P* = 0.87), age (*P* = 0.10), educational level (*P* = 0.07), or SES group (*P* = 0.30) compared to AYAHIV− (**Table 1**).

**Table 1:** Sociodemographic characteristics and questionnaire descriptive statistics of the adolescents and young adults involved in the baseline assessment distinguished by HIV serostatus

	AYAHIV− (n = 193)	AYALHIV (n = 161)	Overall (n = 354)
**Sex**			
Female	99 (51.3%)	81 (50.3%)	180 (50.8%)
Male	94 (48.7%)	80 (49.7%)	174 (49.2%)
**Age group**			
Adolescent (15–19 y)	108 (56.0%)	80 (49.7%)	188 (53.1%)
Young adult (20–24 y)	85 (44.0%)	81 (50.3%)	166 (46.9%)
**School (N/A = 1)**			
None	7 (3.6%)	8 (5.0%)	15 (4.2%)
Primary	2 (1.0%)	5 (3.1%)	7 (2.0%)
Secondary	161 (83.4%)	133 (82.6%)	294 (83.1%)
Secondary technical	15 (7.8%)	5 (3.1%)	20 (5.6%)
University	8 (4.1%)	9 (5.6%)	17 (4.8%)
**Job (N/A = 4)**			
None	171 (88.6%)	133 (82.6%)	304 (85.9%)
Farmer	1 (0.5%)	3 (1.9%)	4 (1.1%)
Self-employment	11 (5.7%)	15 (9.3%)	26 (7.3%)
Employee	2 (1.0%)	4 (2.5%)	6 (1.7%)
Public employee	6 (3.1%)	4 (2.5%)	10 (2.8%)
**SES group**			
Low	69 (35.8%)	49 (30.4%)	118 (33.3%)
Middle	59 (30.6%)	59 (36.6%)	118 (33.3%)
High	65 (33.7%)	53 (32.9%)	118 (33.3%)
**MAKS score, x̄ (SD)**	44.1 (5.1)	44.9 (4.5)	44.5 (4.8)
**CAMI27 score, x̄ (SD)**			
Overall	88.6 (8.3)	90.3 (8.6)	89.4 (8.5)
Factor 1	47.8 (7.0)	47.7 (6.9)	47.8 (7.0)
Factor 2	40.8 (7.9)	42.6 (7.9)	41.6 (7.9)
**TAS score, x̄ (SD)**			
Overall	57.7 (9.6)	56.8 (10.5)	57.3 (10.0)
Factor 1	19.8 (5.7)	20.0 (6.2)	19.9 (5.9)
Factor 2	15.2 (3.7)	14.8 (3.9)	15.1 (3.8)
Factor 3	22.6 (4.1)	21.9 (4.7)	22.3 (4.4)

AYAHIV− – adolescents and young adults without HIV, AYALHIV – adolescents and young adults living with HIV, CAMI – Community Attitudes toward People with Mental Illness, MAKS – Mental Health Knowledge Schedule, SD – standard deviation, SES – socioeconomic status, TAS – Toronto Alexithymia Scale, x̄ – mean

The mean scores on the MAKS and CAMI27 questionnaires were 44.5 (SD = 4.8) and 89.4 (SD = 8.5), respectively. Eighty-nine (25.1%) AYAs had good knowledge of mental health, while most (n = 265, 74.9%) had a suboptimal level. The mean score at the TAS questionnaire was 57.3 (SD = 10.0); 70 (19.8%) AYAs had a score suggestive of alexithymia, while most (n = 160, 45.2%) scored negative.

We found no statistical differences in the MAKS score at baseline based on sex (*P* = 0.55), age (*P* = 0.21), HIV serostatus (*P* = 0.14), educational level (*P* = 0.15), or SES group (*P* = 0.59). Baseline CAMI27 score was higher in AYALHIV compared to AYAHIV− (*P* = 0.041), in those with higher educational level (*P* < 0.001), or in the higher SES group (*P* = 0.042). Men had a lower total TAS score than women (*P* = 0.002), as did young adults (YAs) compared to adolescents (*P* = 0.048).

### Post-intervention evaluation

Of the 354 AYAs enrolled, 315 participated in the psychodrama sessions, with an average participation rate of 94.4% sessions (average of 7.5 sessions, SD = 0.9). After applying our exclusion criteria, we included 281 AYAs in the post-intervention assessment (Figure S1 in the **Online Supplementary Document**). Overall, the mean scores on the MAKS, CAMI27, and TAS after the intervention were 47.1 (SD = 5.3), 88.9 (SD = 10.7), and 54.4 (SD = 11.4), respectively.

**Figure 2:**
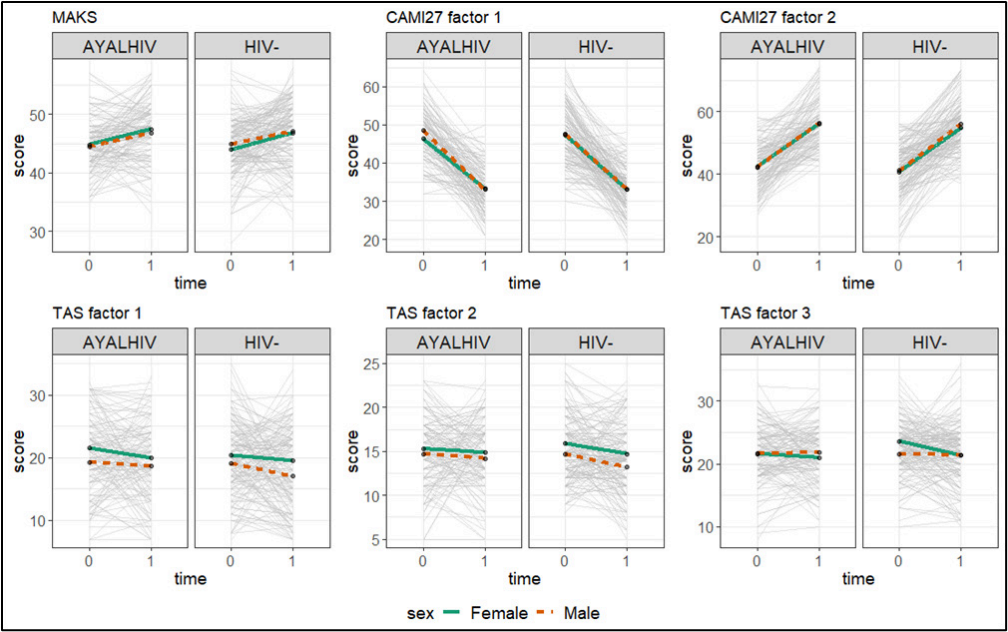
Mean scores for the MAKS, for factors 1 and 2 of the CAMI27, and at factors 1, 2, and 3 of the TAS in the baseline (time = 0) and in the post-intervention assessment (time = 1), as well as stratified by HIV serostatus and sex. The grey lines represent the individual values. AYALHIV – adolescents and young adults living with HIV.

In the RMANOVA model, the MAKS score significantly improved from baseline (44.5; 95% CI = 44.0–45.0) to post-intervention (47.1; 95% CI = 46.4–47.7, *P* < 0.001), without statistically significant differences based on sex, age, or HIV serostatus (**Figure 2**, **Table 2**).

**Table 2:** Descriptive and test statistics from the three repeated measure ANOVA model, with total MAKS, CAMI27, and TAS scores as dependent variables and with one within-subject factor (time) and three between-subject factors (sex, age, and HIV serostatus)

	Descriptive statistics					Test statitsics		
**Sex**	**HIV serostatus**	**Age**	**Time**	**n**	**Mean (95% CI)**	**ANOVA-type statistic**	**Test statistic**	***P*-value**
**MAKS questionnaire**								
Woman	Negative	adol	pre	45	43.9 (39.8–48.0)	sex	0.030	0.86
Woman	Negative	adol	post	45	47.5 (43.2–51.9)	HIV	0.304	0.58
Woman	Negative	YA	pre	32	44.0 (38.2–49.9)	sex:HIV	1.799	0.18
Woman	Negative	YA	post	32	45.9 (38.6–53.3)	age	0.889	0.35
Woman	Positive	adol	pre	29	45.3 (40.1–50.5)	sex:age	1.158	0.28
Woman	Positive	adol	post	29	48.1 (42.2–54.0)	HIV:age	0.797	0.37
Woman	Positive	YA	pre	33	44.4 (39.9–48.9)	sex:HIV:age	1.510	0.22
Woman	Positive	YA	post	33	46.9 (42–51.8.0)	time	37.588	<0.001
Man	Negative	adol	pre	38	44.8 (40.8–48.9)	sex:time	0.299	0.59
Man	Negative	adol	post	38	48.1 (43.8–52.4)	HIV:time	0.001	0.98
Man	Negative	YA	pre	39	45.0 (41.7–48.2)	sex:HIV:time	0.008	0.93
Man	Negative	YA	post	39	46.2 (42.4–50.0)	age:time	0.637	0.43
Man	Positive	adol	pre	33	44.4 (40–48.80)	sex:age:time	0.232	0.63
Man	Positive	adol	post	33	46.0 (40.6–51.3)	HIV:age:time	2.230	0.14
Man	Positive	YA	pre	32	44.7 (39.6–49.7)	sex:HIV:age:time	0.385	0.54
Man	Positive	YA	post	32	47.7 (42.1–53.2)			
**CAMI27 questionnaire**								
Woman	Negative	adol	pre	45	88.2 (81.8–94.5)	sex	1.228	0.27
Woman	Negative	adol	post	45	87.2 (80.0–94.4)	HIV	1.309	0.25
Woman	Negative	YA	pre	32	88.2 (80.6–95.7)	sex:HIV	0.007	0.93
Woman	Negative	YA	post	32	88.8 (78.7–98.9)	age	1.911	0.17
Woman	Positive	adol	pre	29	87.5 (77.8–97.3)	sex:age	0.317	0.57
Woman	Positive	adol	post	29	87.9 (72.6–103.1)	HIV:age	3.272	0.07
Woman	Positive	YA	pre	33	90.0 (81.1–98.8)	sex:HIV:age	0.594	0.44
Woman	Positive	YA	post	33	90.8 (81.4–100.1)	time	0.033	0.86
Man	Negative	adol	pre	38	88.9 (81.7–96.1)	sex:time	0.203	0.65
Man	Negative	adol	post	38	90.7 (81–100.3)	HIV:time	0.208	0.65
Man	Negative	YA	pre	39	88.5 (79.7–97.3)	sex:HIV:time	0.942	0.33
Man	Negative	YA	post	39	87.9 (78.4–97.4)	age:time	0.005	0.94
Man	Positive	adol	pre	33	89.3 (81.2–97.4)	sex:age:time	0.585	0.45
Man	Positive	adol	post	33	87.8 (76.5–99.1)	HIV:age:time	0.036	0.85
Man	Positive	YA	pre	32	92.4 (83.6–101.3)	sex:HIV:age:time	0.337	0.56
Man	Positive	YA	post	32	77.8 (65.0–90.7)			
**TAS questionnaire**								
Woman	Negative	adol	pre	45	56.9 (49.4–64.5)	sex	8.399	0.004
Woman	Negative	adol	post	45	51.6 (43.1–60.1)	HIV	0.493	0.48
Woman	Negative	YA	pre	32	51.9 (45.2–58.6)	sex:HIV	0.913	0.34
Woman	Negative	YA	post	32	49 (38.2–59.7)	age	3.334	0.07
Woman	Positive	adol	pre	29	54.5 (41.7–67.3)	sex:age	0.141	0.71
Woman	Positive	adol	post	29	50.4 (34.8–66)	HIV:age	0.068	0.79
Woman	Positive	YA	pre	33	52.4 (43.1–61.7)	sex:HIV:age	3.232	0.07
Woman	Positive	YA	post	33	51.2 (41.3–61)	time	13.678	<0.001
Man	Negative	adol	pre	38	50.6 (41.1–60.2)	sex:time	0.480	0.49
Man	Negative	adol	post	38	45.6 (35.1–56)	HIV:time	1.896	0.17
Man	Negative	YA	pre	39	49.9 (41.8–58)	sex:HIV:time	0.182	0.67
Man	Negative	YA	post	39	47.5 (36.7–58.4)	age:time	1.803	0.18
Man	Positive	adol	pre	33	52.5 (42.6–62.3)	sex:age:time	0.139	0.71
Man	Positive	adol	post	33	51.4 (41.3–61.5)	HIV:age:time	0.091	0.76
Man	Positive	YA	pre	32	48.7 (35.5–61.9)	sex:HIV:age:time	0.214	0.64
Man	Positive	YA	post	32	47.9 (35.2–60.7)			

adol – adolescent, ANOVA – analysis of variance, CAMI – Community Attitudes toward Mental Illness, MAKS – Mental Health Knowledge Schedule, TAS – Toronto Alexithymia Scale, YA – young adult

The CAMI27 score showed no significant variation from pre-intervention (89.1; 95% CI = 88.1–90.1) to post-intervention (88.9; 95% CI = 87.7–90.2, *P* = 0.86), nor based on the between-subject factors (**Table 2**). Considering the two factors of the CAMI27, the first (‘tolerance and support in the community’) decreased from 47.5 (95% CI = 46.7–48.2) to 33.2 (95% CI = 32.5–33.8, *P* < 0.001). Conversely, factor 2 (‘prejudices and exclusion’) significantly increased from 41.6 (95% CI = 40.7–42.5) to 55.8 (95% CI = 54.8–56.7, *P* < 0.001). We saw no differences in the two factor pre- and post-intervention based on sex (*P* = 0.39), age (*P* = 0.45), or HIV serostatus (*P* = 0.89).

The total TAS score decreased from 57.3 (95% CI = 56.1–58.5) at baseline to 54.3 (95% CI = 53.0–55.6, *P* < 0.001) in the post-intervention evaluation. We also confirmed the difference found at baseline by sex, with men having lower TAS scores overall. We found no statistically significant interactions between time and sex, age, or HIV serostatus (**Table 2**). Considering the three factors of the TAS questionnaire, the first factor (‘difficulty identifying feelings’) and second (‘difficulty describing feelings’) contributed to the decrease (*P* = 0.021 and *P* = 0.015, respectively), while the third (‘externally-oriented thinking’) showed no statistically significant changes (*P* = 0.15).

### Semi-structured interview analysis

We analysed thirty-seven semi-structured interviews with 16 women (43.2%) and 15 AYALHIV (40.5%). The median age was 20 years (IQR = 15–22). The interviews lasted between 20 and 35 minutes.

We coded 472 themes that we found to be relevant; 356 (75.4%) were related to the impact of the intervention and 116 (24.6%) to acceptability/feasibility. The interrater agreement over the main area was very good (α = 0.85; 95% CI = 0.73–0.96), while the agreement over sub-areas was good (α = 0.71; 95% CI = 0.52–0.90) (**Table 3**).

**Table 3:** Frequency of the main areas and sub-areas retrieved in the inductive content analysis of the semi-structured interviews, distinguished by HIV serostatus, sex, and age group*

	AYAHIV− (n = 302)	AYALHIV (n = 170)	Woman (n = 198)	Man (n = 274)	Adolescent (n = 187)	YA (n = 285)	Overall (n = 472)
**Peer-to-peer support**	69 (30.7)	39 (29.8)	39 (25.2)	69 (34.3)	41 (29.1)	67 (31.2)	108 (30.3)
Sharing and self-expression	30 (13.3)	16 (12.2)	17 (11.0)	29 (14.4)	19 (13.5)	27 (12.6)	46 (12.9)
Belonging and trust	21 (9.3)	19 (14.5)	14 (9.0)	26 (12.9)	16 (11.3)	24 (11.2)	40 (11.2)
Interpersonal learning	18 (8.0)	4 (3.1)	8 (5.2)	14 (7.0)	6 (4.3)	16 (7.4)	22 (6.2)
**Social empowerment**	50 (22.2)	38 (29.0)	36 (23.2)	52 (25.9)	37 (26.2)	51 (23.7)	88 (24.7)
Communication skills	22 (9.8)	19 (14.5)	15 (9.7)	26 (12.9)	18 (12.8)	23 (10.7)	41 (11.5)
Mental health awareness	15 (6.7)	17 (13.0)	17 (11.0)	15 (7.5)	11 (7.8)	21 (9.8)	32 (9.0)
Prosocial attitude	13 (5.8)	2 (1.5)	4 (2.6)	11 (5.5)	8 (5.7)	7 (3.3)	15 (4.2)
**Resilience**	60 (26.7)	22 (16.8)	38 (24.5)	44 (21.9)	29 (20.6)	53 (24.7)	82 (23.0)
Coping	30 (13.3)	9 (6.9)	19 (12.3)	20 (10.0)	16 (11.3)	23 (10.7)	39 (11.0)
Breaking habits	20 (8.9)	6 (4.6)	12 (7.7)	14 (7.0)	12 (8.5)	14 (6.5)	26 (7.3)
Self-efficacy	10 (4.4)	7 (5.3)	7 (4.5)	10 (5.0)	1 (0.7)	16 (7.4)	17 (4.8)
**Emotional skills**	46 (20.4)	32 (24.4)	42 (27.1)	36 (17.9)	34 (24.1)	44 (20.5)	78 (21.9)
Empathic listening	20 (8.9)	11 (8.4)	18 (11.6)	13 (6.5)	12 (8.5)	19 (8.8)	31 (8.7)
Emotional intelligence	18 (8.0)	12 (9.2)	19 (12.3)	11 (5.5)	16 (11.3)	14 (6.5)	30 (8.4)
Perspective taking	8 (3.6)	9 (6.9)	5 (3.2)	12 (6.0)	6 (4.3)	11 (5.1)	17 (4.8)
**Acceptability/feasibility**	n = 77	n = 39	n = 43	n = 73	n = 46	n = 70	n = 116
Perceived scalability	26 (33.8)	17 (43.6)	13 (30.2)	30 (41.1)	18 (39.1)	25 (35.7)	43 (37.1)
Affective attitude	28 (36.4)	12 (30.8)	19 (44.2)	21 (28.8)	18 (39.1)	22 (31.4)	40 (34.5)
Practicality	13 (16.9)	6 (15.4)	6 (14.0)	13 (17.8)	6 (13.0)	13 (18.6)	19 (16.4)
Burden	10 (13.0)	4 (10.3)	5 (11.6)	9 (12.3)	4 (8.7)	10 (14.3)	14 (12.1)

AYAHIV− – adolescents and young adults without HIV, YA – young adult, AYALHIV –adolescents and young adults living with HIV

*Presented as n (%) unless specified otherwise.

The most frequent retrieved area was ‘Peer-to-peer support’ (30.3%). We found no statistically significant differences in the frequency of the four main area based on sex (*P* = 0.10), age (*P* = 0.69), or HIV serostatus (*P* = 0.13). In this area, ‘sharing and self-expression’ and ‘belonging and trust’ were the two more frequently reported sub-areas. They referred to the intimate bond between group members created by sharing personal stories and to the empowerment resulting from being able to express hidden parts of one's inner world in a group atmosphere characterised by acceptance and appreciation of each member.

I used to be someone who didn't know how to share my own personal stories with others, I didn't like to share the problems I have in my life, and I've learnt to share my problems with other people*. – Woman, YAHIV−.*

For me, being part of this group has been a privilege. Since then, I've learnt a lot of good things and before I was afraid and sad, now I feel accepted. *– Woman, YAHIV−.*

The group-based method was also important because it gave AYA the possibility to gain new insights through the process of interaction with others and to discover that one's own experience may be shared by many other group members.

That's when we had the face-to-face game where we told each other about what we were going through and I discovered that in the group there was someone who was going through the same situation as me, and when we saw each other, we realized that we were going through the same thing. I was very touched and realized that I'm not alone*. – Woman, AHIV−.*

The second area we identified was ‘social empowerment’ (24.7%). It encompassed the sub-areas of ‘communication skill’ and ‘prosocial attitude,’ referring to increasing skills in positive relationship building, group cooperation, and empathic communication.

Looking at it with the knowledge I have now, my actions would be different: in fact, I could get close to the person and listen to them and know how to be there for them, not keep them at a distance, but make them understand that they can be sincere and upfront*. – Woman, YAHIV−.*

Another important subarea in this area was ‘mental health awareness,’ expressing how the AYAs involved in the sessions gained increased awareness about the importance of mental health as an integral component of human well-being, alongside knowledge on both protective and risk factors in mental health. This also allowed them to develop a non-stigmatising attitude towards people suffering from mental illness.

What I would do at the moment would be to talk to the person and understand what they are feeling, and then I would recommend psychological counselling*. – Woman, YAHIV−.*

Empathise with people suffering from mental disorders: they too are people and deserve love, and I would not want them to be victims of bad behaviour*. – Man, YAHIV.*

The ‘resilience’ area expressed how the intervention helped AYAs improve their capacity for self-regulation and self-care in difficult situations by giving them a better ability to recognise and activate one's own resources, namely ‘coping’ (11.0%), ‘breaking habits’ (7.3%) and ‘self-efficacy’ (4.8%). In particular, ‘breaking habits’ referred to stepping out of one's stereotyped way of acting and thinking in order to develop new and more appropriate ways of handling situations and conflicts.

Before I started taking part in this psychodrama in the past, I had many frustrations and I didn't know how to deal with my emotions and I easily lost hope in believing and living, unlike today when I know how to manage my emotions*. – Woman, ALHIV.*

When I started coming here, I began to open up and I changed my way of being and seeing things a lot and I realized that I am very important myself and I deserve to be good with myself*. – Woman, ALHIV.*

In the ‘Emotional skills’ area, the two most represented were ‘emotional intelligence’ and ‘empathic listening.’ The first included the process of recognising one's own and others emotional state and of using language as a tool to express feelings; the second referred to a mindful, non-judgemental, quality listening aimed at truly understanding what another person is experiencing.

The difference is that if you look around the neighbourhood and within the family, people don't have the spirit of listening, sometimes people judge us without realizing the root cause of why we're acting the way we are. I've learned that you need to be someone who is caring, someone who is ready to listen to the other person's situation so that you can help, there's no way you can help without listening to the people*. – Woman, YAHIV−.*

I have the right to express my emotions and my needs*. – Man, ALHIV.*

Regarding acceptability and feasibility, 14 (37.8%) and 19 (51.4%) AYAs did not report any difficulties nor anything to improve, respectively. Among the experienced difficulties, 14 (12.1%) were classified as a ‘burden’ and concerned the problem of transport for the AYAs who lived more distant or had financial difficulties in paying for it. The ‘practicality’ sub-area was retrieved in 19 (16.4%) themes and related mainly to the initial difficulty in talking about oneself and in getting into another person's point of view.

The ‘perceived scalability’ was mentioned 43 times (37.1%) and referred to both to the willingness to continue the sessions and to extend the intervention to other groups involving more AYAs. In 38 of 40 (95.05%) themes, a positive ‘affective attitude’ was stated, while 29 (78.4%) of the respondents did not express any discomfort during the sessions.

## DISCUSSION

In this study, we assessed the impact and acceptability/feasibility of a community-based psychodrama intervention on AYAs. Both the total MAKS (measuring knowledge on mental health) and TAS scores (measuring level of alexithymia) significantly improved after the intervention, as did the ‘prejudices and exclusion’ factor of the CAMI27. These effects remained significant even when adjusting for sex, age, and HIV serostatus, showing that this intervention can be useful in these at-risk populations. The AYAs found the intervention a useful and pleasant experience; they would have continued it afterwards and would like it to be extended to more people. Adherence to the psychodrama sessions was very good, with more than 94% of participants attending. The main difficulty they mentioned was transportation and its cost for people coming from farther away.

The available scientific literature describes psychodrama interventions carried out in Europe, North America, South America, and Asia [28]. This method has shown its effectiveness and positive impact on mental health in a wide range of applications, ranging from therapy groups with clinical and subclinical patients suffering from different psychological disorders to community-based mental health care programmes, or to personal development plans aimed at, for example, improving socio-emotional skills or changing attitudes. The heterogeneity of the applications and issues addressed indicates that psychodrama can improve the symptoms associated with a wide range of problems and that it is suitable for adaptation to different cultural and social contexts.

Community-based interventions are a key pillar to delivering mental health promotion, prevention, and care. As outlined in WHO's Community Toolkit mhGAP, it is low-cost and can reach many people at their places of residence and work, providing a full spectrum of mental health interventions and reducing stigma [29]. Despite only limited studies, especially for LMICs, there is some evidence that community-based interventions can improve emotional health and mental health literacy [30]. Compared to most psychoeducational intervention methods implemented in the context of LMICs, which are primarily based on frontal lecture modes, the use of psychodrama techniques and group dynamics allows AYAs to get involved directly and personally, thus enabling an experiential learning process and a constructive change in the person's perceptions and functioning. Compared to interventions carried out within hospitals or psychology and psychiatry units, community-based interventions make it possible to precisely reach those people who suffer most from barriers related to stigma, cultural preconceptions, and economic status.

The results of this study showed that mental health knowledge and attitudes improved significantly after the intervention, along with the participants’ ability to identify and express emotions. Surprisingly, we found the ‘tolerance and support in the community’ factor of the CAMI27 to be worse after the intervention. This could be related to the fact that it contains some items related to the medicalisation of patients with mental illness and that the psychodrama sessions emphasised the importance of seeking help from mental health professionals in health centres, which may have led to this counterintuitive result. In a setting where stigma and the use of traditional medicine practices remain widespread, proper information and fostering help-seeking by specialised personnel (i.e. psychologists and psychiatrists) is still a necessary focus [31]. Moreover, in the ‘mental health awareness’ sub-area of our semi-structured interview analysis, the AYAs showed a greater sensitivity and sense of inclusion toward people with mental illness that was paralleled by an understanding that it is important to counsel them or refer them to seek professional support at health units. With a treatment gap estimated at 85% in LMIC, promoting dialogue about mental health (thereby reducing stigma), alongside increasing help-seeking behaviour, community ownership, and responsibility for good mental health, are key steps to improving the mental health of the community itself [32].

Many factors contribute to the promotion of mental health; among them, social and emotional learning is recognised to be pivotal in adolescents [33]. Indeed, this age period is characterised by major hormonal and physical changes that are coupled with a substantial change in the social environment. In this context, the peer network plays a crucial role in the adolescent's life and well-being [34]. The need to improve social and emotional learning is also recognised by adolescents themselves in LMICs; in a focus group study, adolescents identified developing social-emotional skills, especially in interpersonal relationships, as their main need toward good mental health [35].

In this context, psychodrama proved to be a useful approach to improve social and emotional learning. In the semi-structured interviews, AYAs stated that the participation in these sessions enhanced their sharing and self-expression ability and communication skills, resulting in two main areas – peer-to-peer support and social empowerment. Considering the 12 sub-areas we identified in our content analysis, they fall within the society-level actions required by the ‘helping adolescents thrive toolkit’ proposed by WHO to improve adolescent mental health [7]. These are interpersonal, emotional regulation, and higher-order thinking skills (e.g. decision-making, problem-solving), self-esteem, and coping. According to the sub-areas found in the semi-structured interviews, psychodrama sessions influenced all of the above dimensions. These dimensions act in the outermost level of the social-ecological system according to Bronfenbrenner's theory, meaning on the individual as they are embedded in a specific sociocultural environment. This can create an environment that enables promotion and prevention in mental health, leading to the concept of ‘community mental health competence’ [36]. Mental health is indeed a shared resource promoted by a competent community. These psychodrama sessions enabled AYAs to gain knowledge, practical skills, and empowerment in actively taking care of their own mental health and in creating relational contexts in their community that promote it. Through this intervention, we intended to promote AYAs as actors taking an active role in promoting their mental health and that of their community. Indeed, the empowerment of adolescents is recognised as fundamental to their mental health and resilience, and it promotes social inclusion and harmony [28]. Available evidence supports the role of community-based psychodrama in improving personal skills such as empathy, self-awareness, increasing social skills, and understanding one’s strengths and weaknesses, thus fostering emotional and cognitive integrity [37]. Furthermore, psychodrama has been found to address well-being and hopelessness, with a positive effect on both. Hopelessness is a particularly important aspect to consider in Mozambique, which is among countries with the highest incidence of suicide deaths in sub-Saharan Africa, as it is believed to be one of the predictors of suicide [38]. Improving social and emotional learning is one of the most effective strategies recognised globally for suicide prevention and suicide risk reduction at community level [8,39].

We found no differences in the effect of the psychodrama intervention based on HIV serostatus. Increased self-connection and emotional awareness and the creation of a group feeling free from judgement (and thus stigma) is particularly helpful for AYALHIV, as it creates a feeling of acceptance within themselves and by others [40]. Furthermore, integrating mental health interventions into those for HIV has been shown to have mutually beneficial effects both on mental health itself and on improving adherence to antiretroviral therapy with better quality of life [41].

The psychodrama intervention proposed here was well-accepted by the participants, with a high participation rate (approx. 94%). The greatest burden was related to transport and its cost for those who lived far away. The early difficulties in practising some techniques, such as the double and the role reversal, were overcome over time, and the interviews suggested that AYAs were willing to continue the course and extend it to other AYAs because of its positive effect on the community. The challenge of adopting an intervention model developed in a different cultural context was also overcome through the sessions being held by the CHWs. This made it possible to have group leaders embedded in the local sociocultural background and to reach more AYAs at a lower cost. The employment of CHWs is a key strategy for the large-scale implementation of culturally oriented mental health interventions [30].

This study has some limitations. First, it was monocentric, conducted only in the city of Beira, so it is not representative of the whole national context. Second, although all three questionnaires have been previously used in sub-Saharan Africa and LMICs, no cultural validation was conducted in Mozambique, so some of the items may not be fully appropriate for this setting. Moreover, the absence of a control group does not allow the entire effect observed to be attributed to the intervention. Finally, the open-ended nature of semi-structured interviews may lead the interviewer to steer the questions, with consequent observer bias, while the respondents may attempt to answer in the way they think is acceptable, leading to a social desirability bias. However, the use of a mixed methodological approach was useful in limiting the biases arising from both the use of the questionnaires and the qualitative content analysis of the interviews.

The use of psychodrama and action methods is effective in raising awareness about mental health. Through specific strategies based on group dynamics work and participatory methods, AYAs can improve their socioemotional functioning and empower their resilience and coping strategies toward major mental health stressors. These kinds of interventions can be well accepted and well-integrated into the LMIC context thanks to a culturally oriented approach and the involvement of the community.

## CONCLUSIONS

This psychodrama intervention proved to be effective in enabling AYAs with better knowledge about mental health and improving their ability to recognise and describe emotions. This may improve related outcomes in AYAs and spread a positive culture towards mental health. The intervention was equally effective in AYALHIV or AYAHIV−, making it useful for promoting mental health in such an at-risk group. The use of CHWs helped us culturally adapt the intervention method developed in a different context, resulting in good acceptability and feasibility. Consequently, the AYAs showed willingness to continue the sessions and extend them to more people. It is important to continue to foster community interventions that address the social and emotional learning of the AYAs to create a competent community that can promote mental health and care for its members by serving as an additional preventive instrument for related disorders and suicidal risk.

## Additional material


Online Supplementary Document
