# Supplementary material for: Assessing the impact of a community-based psychodrama intervention on mental health promotion of adolescents and young adults in Mozambique: A mixed-methods study
Source: J Glob Health. 2024 Jul 26;14:04182. doi: 10.7189/jogh.14.04182 (PMC11271167; doi:10.7189/jogh.14.04182)
Supplement: Online Supplementary Document [file jogh-14-04182-s001.pdf]

Online Supplementary Document

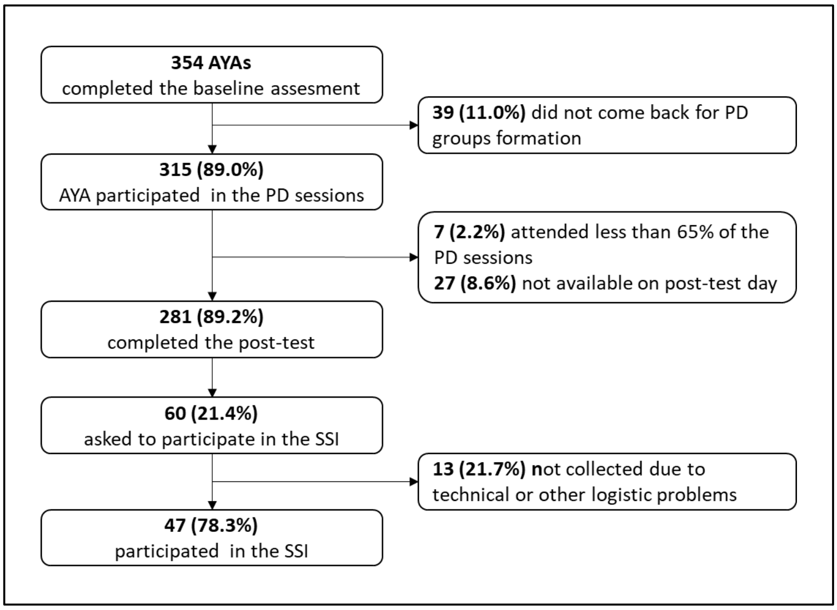

Figure S1 - Flowchart of the adolescent and young adults (AYA) included in the study. PD: Psychodrama. SSI: Semi-structured Interview.

Classification index code for the Semi structured Interviews

AREA	SUBAREA	Definition	Examples
**SOCIAL EMPOWERMENT**	**MENTAL HEALTH AWARENESS**	With this code we have labelled phrases that refer to the acquiring of new knowledge and awareness about mental health and its importance as an integral component of a human wellbeing. Included here are phrases showing knowledge of the most important protective and risk factors for mental health as well as an inclusive and non-stigmatizing attitude towards people suffering from mental illness.	"*In my opinion to be mentally well you need to feel comfortable with yourself, you need to feel accepted and not excluded. That's why, in order to be well, you need to have around you people who can support you regardless of the situation*." "If I am near a person with a mental health problem, I will try to get close to them to try to understand what is really happening to them, if I can help them, and if I can't, I will go to the health unit."
	**PROSOCIAL ATTITUDE**	With this code we have labelled all phrases referring to the experience of being able to offer something useful to another person and the phrases referring to the interest in the well-being of others.	*"It inspired me to have the opportunity to help someone, to do something to motivate and help someone who is going through a difficult time". "if I meet someone in pain, I want to create an environment where they can feel accepted".*
	**COMMUNICATION SKILLS**	With this code we have labelled phrases referring to increasing skills in positive relationship building, group cooperation and empathic communication.	*"This course helped me to be able to understand people, to be able to connect with someone, to be able to talk about what is really meaningful". "I noticed a huge difference because in my neighborhood people do not respect each other much, whereas here there is a lot of mutual respect and understanding. Here when a person speaks, others listen and are careful to understand what the person has to say, unlike in the neighborhood".*
**PEER-TO-PEER SUPPORT**	**BELONGING AND TRUST**	With this code we have labelled phrases referring to group cohesion, intended as a sense of acceptance and appreciation of each member of the group, and mutual trust, meaning an atmosphere of intimacy and security. Included here are phrases referring to having found support and a reference point in other group members or in the trainers.	*"My colleague opened up to me and the most touching thing was that I felt privileged when he said that what I am about to tell you I have never told anyone in my house". "I felt comfortable and fearless because I knew there would be confidentiality about the things I told". "The difference from the neighborhood is that here we all felt comfortable, no matter where we came from, and we were always there as a family, without judging people or discriminating, and we were all there in the same boat". "My trainer has always supported me in overcoming my barriers".*
	**INTERPERSONAL LEARNING**	With this code we have labelled phrases that refer to the acquirement of new understandings and insights through the process of interaction with others. Included in this code is both the experience of enriching oneself through the confrontation with very diverse people as well as the recognition that one's own experience may be an universal human experience or at least shared by other group members	"That's when we had the face-to-face game where we told each other about what we were going through and I discovered that in the group there was someone who was going through the same situation as me… and when we saw each other we realized that we were going through the same thing... I was very moved and realized that I'm not alone". "These sessions have transformed my way of being and thinking because I've been able to socialize with people of different ages, people who have made me understand how to deal with different situations in life. That's why I'm speechless and very grateful".
	**SHARING AND SELF EXPRESSION**	With this code we have labelled phrases that refer to the opportunity to open up to each other and to share life experiences in the group. Included here are phrases that express the sense of release in being able to give voice to one's experiences and committing one's emotions to the group. Also included here are phrases that indicate shedding one's own masks and inhibitions in order to show oneself authentically to others.	*"My challenges consisted of opening up memories of things from my past that I didn't want to talk about because they were things I wanted to forget... but in the end I freed myself and, believe me, I told them, and now I feel free and at peace with myself". "One thing I discovered that I didn't know about myself is the issue of talking and sharing... I used to be a very lonely person and not very friendly, but when I started to participate, I started to open up and to talk often and tell my thoughts and problems". "In one of the sessions a colleague told me the secrets of his life which by the way were things he had experienced just this year… It was wonderful because I felt that he had confided in me and shared his struggles and frustrations with me, and I want to believe that he also felt good talking to me". "This course helped us to free ourselves and express ourselves... here is a great place to be free to express ourselves at all times and there were moments when we cried with relief".*

**EMOTIONAL SKILLS**	**EMPATHIC LISTENING**	With this code we have labelled phrases that refer both to the experience of listening to another person and being listened to by someone else, without interruption and without judgement. The emphasis here is placed on a mindful and compassionate quality of listening, aimed at truly trying to understand what another person is saying and experiencing.	*"When I spoke to my group partner, she was very patient and did not intervene at all, and it was gratifying to know that there is someone who can listen to you without criticizing you"* *"Compared to the neighborhood, the way I converse here in this group is very different because this group taught me to listen more, to be more attentive and to understand what the other person is saying or can say to someone. Basically, there's a huge difference compared to the neighborhood"* *"If I met a person suffering from a mental health problem I would try to understand them, I would be very calm and patient in listening to them to try to be in their shoes to understand them more deeply".*
	**EMOTIONAL INTELLIGENCE**	With this code we have labelled phrases referring to the process of recognizing, describing and interpreting one's own and others' feelings. This includes phrases referring to: distinguish one's own emotional states, observe how emotions manifest themselves in one's body, identify which thoughts and needs determine one's emotions, use language as a tool to express feelings.	*"I discovered that inside me there are different emotions at different times and that depending on my emotions I make actions that I was previously unaware of or did not pay attention to"* *"The first thing to do was to share with colleagues how I felt about my emotional state and, at the same time, talk about my needs and difficulties"* *"I have been encouraged to explain and to express how I felt about what had happened to me"*
	**PERSPECTIVE TAKING**	With this code we have labelled phrases that refer to the experience of perceptively decentralising oneself from one's own role to assume the perspective of another person, through the psychodramatic technique of role inversion. The experience described here is that of getting into another person's shoes to view the situation from another person's cognitive and emotional perspective.	*"What really made an impact on me was when a colleague told me about something that was happening in his life with his family, and I could see it through his eyes and I was touched"* *"I was very touched to see someone put herself in my place and it was unusual for me to be able to understand and analyze what she was saying"*
**RESILIENCE**	**COPING**	With this code we have labelled phrases referring to increased awareness of one's own resources, understood as the skills or strategies we can mobilise in times of trouble or emotional distress. We have included here the phrases referring to the capacity for self-regulation and self-care.	*"During the sessions we had, I learnt various ways to feel better with myself when I am sad, worried or a bit nervous. Now it doesn't change anything for me whether the sadness comes or goes, because I now have the necessary tools for self-help"* *"One of the resources I learnt to use was relationships: when I was sad, instead of isolating myself, I learnt to seek help by talking to other people to calm myself down. Not only that, but the body is also one of my resources: I walked to relax when I was sad"*
	**BREAKING HABITS**	With this code we have labelled phrases that refer to the experience of leaving behind one's stereotyped behavior and breaking out of one's habitual way of acting and thinking. Included here are all the phrases from which emerges the ability to elaborate new and more appropriate ways of behaving in situations, handling conflicts and making choices.	*"This course helps people to be more open-mind, every time I have come to these psychodrama sessions I have seen many people progress in their mindset"* *"It would be very useful for a teenager to attend this course so that he can reflect on what he does, what he wants and what decisions he has to make"*
	**SELF EFFICACY**	With this code we have labelled phrases that express the strengthening of one's sense of self-efficacy understood as the perception of oneself as a person capable of achieving one's goals, expressing oneself truthfully and effectively, and contributing in a meaningful way to the lives of others.	*"I felt confident and safe, I discovered that there are people who appreciate me and believe in me"* *"I felt very important to know that someone could trust me with their secrets"* *"I remember when I was telling someone else something about my life… I discovered that I had the ability to tell my story to my colleague, I just needed that courage and openness and experience"*

Sociodemographic questionnaire

Section 1: Individual data

1. What year were you born?________Age ___________

2. Neighbourhood where you live: ___________________

3. Gender:
□ Male Women's □ □ I'd rather not answer

3. Marital status:
□ Single □ De facto union
□ Married □ Divorced □ Widower

4. Do you have children?
□ Don't □ 1 □ 2 □ 3 □ 4 or more

5. Education:
□ None
□ Primary education
□ Secondary education
□ Professional technical level
□ Higher Education (university, master's, other)

6. Are you currently a student?
□ Yes □ No

7. Work
□ None
□ Subsistence farming
□ Self-employment (point of sale, informal)
□ Works in the public sector
□ Employment contract (salaried)

8. Do you have a family member, friend or close person who suffers from a mental illness?
□ No □ A family member
□ A friend □ A classmate or co-worker
□ Other

9. Have you accessed any of these services in the past?
□ None □ Psychosocial support
□ Psychiatry □ Psychology Service
If so, why (diagnosis)? __________________________

10. Are you currently counselling in one of these services?
□ None □ Psychosocial support
□ Psychiatry □ Psychology Service
If so, why (diagnosis)? __________________________

Section 2: Information about your home

1. At home there is a floor washable?	□ No<br>□ Yes
2. Is there a bathroom at home?	□ No<br>□ With washable floor (latrine)<br>□ Of perishable material (earth, branches, wood, hole in the ground) or with drain and septic tank
3. At home there is a roof of perishable material (coconut palm leaves, straw)?	□ No<br>□ Yes
4. Running water at home?	□ No<br>□ Yes
5. Electricity or electric generator at home?	□ No<br>□ Yes
6. Do you have a television at home?	□ No<br>□ Yes
7. Do you have a smartphone or a phone?	□ No<br>□ Telephone<br>□ Smartphone
8. Are there means of transport at home? (Select all valid answers)	□ No<br>□ Bicycle<br>□ Motorbike<br>□ Car
9. Your family owns farmland?	□ No<br>□ Yes
9. How many people live in your home?	________
10. How many rooms are in your home?	________

Semi structured Interview Guide

**Main Objective:**<br>To estimate assess the impact of a community-based psychodrama intervention on adolescents and young adults in terms of knowledge and attitude towards MH and on emotional consciousness and skills in Sofala Province, Mozambique.

Stages of the interview:

1. Introduction *(approximately 2 minutes)*
The interviewer introduces the objective and topic of the discussion that will take place during the interview.

2. Discussion *(30 minutes)*
The interviewer asks questions related to the main purpose of the interview. It is at this stage that the most important information is collected.

Questions:

1. What's your name? How old are you?
2. To begin with, tell me about your experience in these psychodrama sessions.
3. Tell me about the moment that marked you the most during these sessions.
4. Tell me about a time when you shared a part of your life with your group. What did that mean to you?
5. Tell me about a time when you learned about something from the personal life (a secret) of another person in your group. What did you feel after learning about it?
6. Do you remember the activities with the cards of emotions and needs? Tell me about something that struck you about emotions and needs.
7. In these encounters, there were many moments that also involved your body, breathing and movement... What marked or touched you and why?
8. Tell me about a moment in these sessions when you discovered something about your life or something about yourself that you didn't know before.
9. What was it like for you to be part of this group? How did you feel?
10. Do you remember the times when you paired up with a group mate to talk about something personal in your life and hear from the other colleague? What marked or touched you and why?
11. What was it like for you to put yourself in your colleague's shoes and feel how they feel?
12. During these sessions, what is the difference you noticed here in the group in the way of talking and listening, compared to the way you were used to it at home or in the neighbourhood?
13. What are the good things about the sessions that you would like to take to your community (family, neighborhood, school, etc.)? What wouldn't you like to bring?
14. Tell me about a time in your past when you felt difficult emotions (sadness, anxiety, anger...) and tell me what you did in that situation.
15. If, in the future, you were to go through a similar situation or even with difficult emotions... What would you do?
16. Imagine yourself in the future next to a person suffering from depression, anxiety, or other mental health difficulty... What would you do?

17. Tell me about a resource you used when you were sad or bad, and a resource you want to use the next time you feel sad or bad. Give me an example.
18. How can these sessions help you in your daily life?
19. In your opinion, do you think it is important for adolescents and young people to take this type of course? If so, why/if not, why?
20. In your opinion, what are the important things to be well mentally/psychologically?
21. Have you always had the same idea about the important things to be mentally well? How do you think these sessions might have changed that idea?
22. It's also important for us to realize the difficult aspects you've experienced... What were the difficulties you encountered during this psychodrama course?
23. During the sessions we had, were there any moments when you felt unwell/uncomfortable? If so, tell me about them.
24. What would you like to see change in this course? How do you think it could be improved?
25. Before I end the interview, is there anything you'd like to talk about?

4. Conclusion *(approximately 2 minutes)*
The interviewer answers any questions, thanks the participants.
